# Psychosocial and cultural aspects of pseudocyesis

**DOI:** 10.4103/0019-5545.42398

**Published:** 2008

**Authors:** Perpetus C. Ibekwe, Justin U. Achor

**Affiliations:** Department of Obstetrics and Gynecology, Ebonyi State University, Abakaliki, Ebonyi State, Nigeria; 1 Federal Neuropsychiatric Hospital, New Haven, Enugu, Nigeria

**Keywords:** Africa, false pregnancy, Nigerian women, partner abuse, pseudocyesis

## Abstract

Though considered rare in the developed countries, pseudocyesis is fairly common in gynecological practices in Africa. Using a case report and an overview of the literature, this paper posits that the elucidation of the psychosocial and cultural contexts within which a given patient lives can provide a basis for the empathic understanding of the reasons for the development of pseudocyesis. The case underscores the contributions of extreme poverty, relationship instability, and recurrent partner abuse in the enactment of pseudocyesis within a culture that treasures children for economic survival and generational continuity. The awareness of this cultural dimension is considered relevant to effective clinical care.

## INTRODUCTION

Although pseudocyesis has been recognized as a clinical syndrome since antiquity, it was only within the last one century that systematic attempts have been made to understand its pathophysiology and etiology. As presently construed, pseudocyesis describes a clinical syndrome in which a woman who is not pregnant manifests a strong conviction of being pregnant in association with the symptoms and signs that mimick the experience of being pregnant.[1,2] Pseudocyesis differs from malingering in which a non-pregnant woman consciously claims that she is pregnant to gain certain advantages. It is also quite different from the delusions of pregnancy found in schizophrenia and related psychotic disorders.[3]

The western medical literature describes pseudocyesis as a very uncommon condition, with a prevalence in contemporary gynecological practice of 1-6 cases per 22,000 births,[4] whereas in Africa, there is a higher prevalence, with estimates of about 1 out of 160 patients presenting for infertility treatment.[5] Generally, the majority of cases of pseudocyesis are observed among women aged 20-44 years, although no age is exempt,[6] 80% of the affected persons are married[7] and both single and multiple episodes of the condition have been described.[2] The symptoms and signs reported in pseudocyesis include amenorrhea, abdominal enlargement, breast enlargement, and changes around the nipples and areolae, uterine enlargement and patients’ reports of fetal movements, as well as constitutional symptoms like nausea, vomiting, weight gain, and reduced appetite.[2,6,8] The usual duration of these symptoms range from a few weeks to 9 months or longer. The diagnosis of the condition is usually based on the finding of a full or distended abdomen with an inverted umbilicus in a patient with a suggestive history as well as through the demonstration of absence of the fetus and placenta by use of abdominal ultrasound.[9]

The etiology of pseudocyesis is still unclear despite considerable medical interest and speculations.[10] Most of the currently accepted causal theories emphasize an interaction between psychologic factors and the reproductive system, probably mediated by hormonal influences. The key psychologic factors that are thought to have etiological significance are of three kinds, *viz*. (1) An intense desire for, wish or fear of pregnancy, which results in pseudocyesis through complex neuroendocrine mechanisms.[6] This appears to explain the development of the condition in the setting of infertility, second marriages, recurrent abortions, after gynecological operations, and to force a marriage.[6] (2) Misperception or misinterpretation of sensory stimuli as being indicative of pregnancy,[7] especially in states of abdominal distention or pressure on the pelvic structures. In susceptible persons, such perceptual aberrations initiate processes that could lead to pseudocyesis via neurochemical and hormonal pathways.[9] For instance, Benzick reported a case of pseudocyesis in a woman who had presented with acute abdominal pain which she had believed was labor pain, but was later diagnosed as acute cholecystitis.[11] A similar mechanism is believed to operate in cases of pseudocyesis observed among patients with pelvic and abdominal tumors, aging, and fluid retention from various sources including oral contraceptives.[6] (3) The condition sometimes develops in the setting of depressive disorder and the complex neuroendocrine changes that accompanies it.[6,9,12,13]

The identified psychologic factors are associated with pseudocyesis in the context of promotive obstetric and/or gynecological challenges like infertility, recurrent abortions, threat of menopause, re-marriage, sterilization surgery, etc.[6] Though the involvement of neuroendocrine mechanisms in the causation of the condition is widely accepted,[10] the mode of interaction between the reproductive target organs and the brain to produce the disorder is not as clear. The hypothesized mechanisms have usually included involvement of the hypothalamus and possibly pituitary with modulation of hormonal output and consequent effects on reproductive target organs that may include anovulatory cycles, corpus luteum hypertrophy, and altered sensitivity to specific hormones, etc.[6,14–16]

Not much is known about the social factors that make the development of pseudocyesis possible. However, Dafallah observed a predominance of women of low socioeconomic status in the series of 20 cases he described,[5] whereas, Koic *et al.* remarked on the over-representation of women of lower educational attainment in the literature of affected persons.[9] Similarly, some authors have underscored the roles of emotional turmoil, childhood sexual abuse, and problems in the relationship with significant persons in the enactment of cases of pseudocyesis.[13,17–19] It is not clear how the psychologic and social factors interact to cause the condition, although the stress response model might appear relevant here.[20]

Kleinman indicated that the illness presented by a given patient has meaning whose understanding is related to providing effective treatment to the patient.[21] The approach to unearthing the often polysemic meaning of each illness experience requires an empathic phenomenologic method which seeks to share the lived world of the given patient to understand the sociocultural and emotional determinants of the illness.[22]

The purpose of this paper is to highlight the importance of the intersection of psychosocial and cultural factors in the enactment of pseudocyesis in the African context.

## CASE REPORT

Ms. M.N., an 18-year-old illiterate slim lady presented to the Accident and Emergency (A&E) Department of Ebonyi State University Teaching Hospital in the company of her boy friend with complaints of intermittent, moderately severe, lower abdominal pain which she insisted was labor pain. She had experienced the pain for 5 h prior to presentation and noted that the pain was associated with spotting per vagina, which to her was confirmatory evidence that she was in labor. She had explained the spotting as “show”. Incidentally, the abdominal pain had started shortly after she had been beaten by her consort.

Subsequently, the patient indicated that she had been having amenorrhea for 8 months prior to the onset of the pain. Before the onset of the amenorrhea, she had been having regular monthly cycles and her menses usually flowed for 5 days since the attainment of menarche at the age of 15 years. The amenorrhea had developed shortly after she had moved in to live with her boyfriend, with whom she had been having regular sexual intercourse.

Four months prior to the current presentation, the patient had visited the General Outpatient Department (GOPD) of the same hospital with complaints of spotting per vagina which had developed after 4 months of amenorrhea, again shortly after a fight with her consort. At that time, she did not have abdominal pain but was convinced that she was having a threatened abortion. She recounted having experienced tiredness, loss of appetite, sleeping excessively, vomiting after food, breast enlargement, and changes around her nipples. She was not sure at that time whether her abdomen was getting enlarged or not and was also not certain whether she had been feeling any fetal movements. She, however, indicated that she had earlier consulted a nurse in the city who assured her that she had “heard” her “baby's heart beats.” The attending doctor at that time had examined her but did not believe she was pregnant. He then asked her to do some investigations: urine pregnancy test, PCV, and abdominal ultrasound and to see him the next week with the results. The patient did not keep that appointment. In the course of the interview however, it was established that the patient's boyfriend had actually given her money for the tests to be conducted, but she never turned up for them.

Clinical assessment at the A & E by the attending resident doctor in Obstetrics showed that Ms. M.N. was in painful distress, with normal respiratory, and cardiovascular systems. Her abdomen was full, firm, and nontender, and the umblicus was inverted. She did not have any abdominal masses and the bowel sounds were normal. Her breasts were of normal size, firm and did not show any nipple or areolar changes. The vulvo-vagina was normal with no overt signs of bleeding. She was very tense, uncooperative, and did not consent to a digital vaginal examination. The rest of the physical examination did not reveal any abnormalities. She was then asked to do an abdominal ultrasound scan. The scan showed a nongravid, empty, anteverted uterus with normal pelvic structures. When the patient was presented with these findings and the conclusion that she was neither pregnant nor in labor, she became very angry, wept uncontrollably and hurled insults at the doctors attending to her.

At this point, the Psychological Medicine Department was consulted. At interview with the resident doctor from Psychological Medicine, the patient noted that she was the second and only daughter in a family of three children of her mother's, who got married into a polygamous home of three wives. Her mother was the second wife. The patient's father had died while she was very young, whereas her mother, who had been her sole source of support, a hardworking farmer, was now incapacitated with “serious eye problem.” The patient did not receive any formal education and had been hawking groundnuts in the village until a year and one-half previously when her stepmother brought her to live with her in the city. On her arrival, the stepmother got the patient a job as a laborer in a quarry plant. The work at the quarry begins early in the morning and does not end till dusk. She described the work as gruesome, tedious, and exerting. It was while working at the quarry site that Ms M.N. met the young man who later became her boyfriend. The said man, 25 years old, also an illiterate, was working as a commercial motorcyclist when their paths crossed. Afterward, they fell madly in love and before long the patient packed her few belongings and moved into his home and started co-habiting with him. Shortly later, she stopped working at the quarry. She was convinced she loved the man and that they were going to get married soon.

Their relationship was passionate, intense, and happy initially. The man was then providing adequately for her material needs. Over time however, things changed. The man's mother learnt of the relationship and came to the city to spend some days with them. During the visit, she told her son that he was not going to marry the patient because she was a stark illiterate. She wanted him to marry an educated lady to compensate for his own illiteracy. Not long after that, the once happy relationship turned sour. Arguments, quarrels, and fights now became regular features of their interaction. Sometimes, the man would beat her and ask her to leave his home. He began telling her explicitly that he was not going to marry her. He stopped caring dutifully for her. Sometimes he would leave the house without making money available for her day's feeding.

While the turmoil raged, the patient's elder brother visited her from the village and in the course of the argument that ensued between them, ordered the patient to leave her boyfriend's home immediately. On her part, Ms M.N. maintained that she was not going to leave because she had become pregnant for him and would have no means of taking care of the child without the support of his father. Moreover, she had strongly believed that when the baby got delivered the man's attitude toward her would improve and his mother would not have any choice but to accept her and the child. It was because of these considerations that the patient resisted attempts by her elder brother to force her home.

The patient's boyfriend pointed out that the main reason for the frequent fights was that he had been trying to persuade her to leave his house without success. This angered him and compelled him to beat her on many occasions.

Ms. M.N.'s mental state examination showed a young lady who looked her stated age, was neatly dressed, looked anxious, and appeared to be in painful distress. At the commencement of the interview, she began crying but calmed down after reassurance and cooperated with the interview. She spoke rapidly but coherently and with normal prosody. With time she became fidgety and reported feeling apprehensive. She did not admit to feeling depressed. Her thought content showed a strong belief in her being pregnant, but this was considered an overvalued idea rather than a delusion because she indicated that if “confirmatory testing” showed that she was not pregnant, she would accept it calmly and afterward leave her boyfriend's home. She did not admit to any hallucinations. The rest of the mental state examination was unremarkable.

Overall, she was thought to have pseudocyesis based on the clinical presentation. Subsequently, she was reassured, informed about the diagnosis and of the need for the initiation of problem-solving counseling sessions the next day. She was also given a prescription for an anxiolytic. On his visit the next morning, the attending doctor learnt that the patient had self-discharged herself the previous evening.

## DISCUSSION

The patient whose case has been reported showed most of the important features of pseudocyesis: conviction of being pregnant in association with amenorrhea and subjective features that mimick true pregnancy. The patient's expectancy of pregnancy was strongly reinforced by having regular sexual activity with her boyfriend and the onset of amenorrhea. In addition, she had this conviction and anticipation for months and presented in false labor. About 1% of the persons presenting with pseudocyesis present with false labor.[8]

The patient's life circumstances reveal a perversive insecurity, powerlessness, and helplessness emanating from the pervasive poverty of her background and her turbulent and abusive relationship with her boyfriend. The pattern of abuses included both the physical and emotional dimensions. These were coupled with the rejection of the patient as unsuitable for marriage first by the mother of the patient's boyfriend and later by the boy himself. This rejection and the patient's tenacious involvement in the relationship were cited as the reasons for the physical and emotional abuses. Also, the cultural practice of relatives wielding a strong influence in the selection of marital partners for their wards certainly contributed to the difficulties that attended the patient's relationship with her boyfriend.

In the throes of the emotional difficulties in which the patient was immersed, fantasies and wishes of becoming pregnant probably developed as a way of achieving fulfillment and stability in the relationship as well as gaining acceptance in the family of origin of her consort. It is likely that the intensity of the ruminations over getting pregnant as a means of overcoming the adverse psychosocial experiences she was involved in contributed to the processes that culminated in the development of a false pregnancy. Pseudocyesis is believed to develop in circumstances in which the wish to get pregnant coexists with ambivalence and fear of pregnancy.[6,9,20]

In Africa and other societies where fertility and childbirth are emphasized and celebrated, it looks as if the feminine identity, self-respect, and fulfillment are measured by the achievement of conception, failing which the individual considers herself a failure or socially incompetent person. In such contexts therefore, the reproductive capacity is treasured for its heuristic value in the social, psychologic, and economic domains,[5] and universal love for and attachment to having children remains a defining characteristic of women's social status in such cultures. In the society in which the patient lives, children are regarded as precious gifts from God which are to be treasured notwithstanding the circumstances of their birth.

Marusiae *et al.* underscored the relevance of marital dissatisfaction and the woman's wish to be loved and appreciated in the development of pseudocyesis.[13] Our patient was rejected as a marital partner by her consort and his mother. This rejection was shown in many ways including deprivation of money for feeding as well as recurrent physical and emotional abuses. Could the woman's wish to be loved in the context of the emotional anguish engendered by an adversarial and unfulfilling relationship contribute to the development of pseudocyesis? Certainly, such a conflictual state can lead to neuroendocrine responses that may contribute to the accompanying stress reaction.[6,20] Koic *et al.* noted that pseudocyesis develops as a response to intense stress in persons who are ambivalent toward pregnancy.[9]

The economic dimension probably contributed significantly to the patient's intense desire for a child. She came from a very poor background. Her mother who had been her source of support had become incapacitated by physical illness and disability. She had moved to the city after years of grinding poverty and privation in the village, in the throes of the adolescent crises of identity, individuation, and autonomy. She had no proper social anchor in the city and disliked the drudgery of working at the quarry. As a culmination of these considerations, she probably longed for marriage as a realistic pathway to assuring her survival, economic sustenance, and having a stable life. It has been argued that economic dependency and financial insecurity make women to endure emotional and physical abuses[23] and that this is perpetuated by low self-esteem and powerlessness on the part of the woman.[24] Each of these factors appeared to have featured in the enactment of this case. It is however not clear to what extent such factors operate in the context of the lives of other persons that suffer pseudocyesis in Africa.

The cultural attachment to childbirth and procreation among Nigerians have been explained as emanating from the multidimensional effects of having children, as economic security especially in old age, for generational continuity and immortality by identification with one's children, and as symbols of social status and achievement. Ladipo had noted that the desire to have children in African cultures exposes women to different forms of stress.[25] Part of this stress has been shown by the patient's readiness to endure the recurrent physical and emotional abuses, with the consolation that she would soon have a child. Does the perception of inherent powerlessness in the context of diminished fecundity lead to the development of pseudocyesis as a compensatory condition? It has been observed that in the context of reduced fecundity, poor and illiterate women, not having the sophistry and diverse repertoire of opportunities for self-advancement available to educated and richer persons might be more likely to develop pseudocyesis.[5] Further studies are required to shed light on this issue.

The patient displayed an intense emotional outburst when she was informed of the negative obstetric findings regarding her being pregnant. The obstetrician's verdict sharply contrasted with her strong convictions and expectations which she had nurtured over several months. Such emotional reactions accord with the experiences of other clinicians.[3,6,11] Psychiatrists’ intervention is often required to effectively manage such challenging crises.[11,13] Otherwise, a therapeutic impasse would ensue. This underscores the necessity for involving psychiatrists in the treatment of cases of pseudocyesis.

It appears as if pseudocyesis develops in a cultural matrix that treasures children and affects individuals who conceive procreation as a symbol of feminine fulfillment and defining identity, who are embroiled in relationships that are mostly characterized by insecurity, dissatisfaction and an intense wish to be loved or whose significant relationships are tumultuous or adversarial. The evaluation of the psychosocial context of patients presenting with pseudocyesis is germane to the delivery of appropriate and effective person-centered care to such patients.
